# Animal-Assisted Psychoeducational Intervention in Paediatric Oncohaematology: Evidence from a Single-Centre Observational Study

**DOI:** 10.3390/children13010136

**Published:** 2026-01-16

**Authors:** Chiara Battaglini, Valentina Isaja, Gaia Riscossa, Mario Giordano, Paola Quarello, Giulia Zucchetti, Franca Fagioli

**Affiliations:** 1 Department of Paediatric Onco-Haematology, Regina Margherita Children’s Hospital, Azienda Ospedaliera Universitaria Città della Salute e della Scienza, 10126 Turin, Italyfranca.fagioli@unito.it (F.F.); 2 Fondazione ZOOM, 10040 Turin, Italy; 3 Department of Sciences of Public Health and Pediatrics, University of Turin, 10123 Turin, Italy

**Keywords:** animal-assisted intervention, paediatric oncology, psychoeducation, emotional regulation, hospitalisation

## Abstract

**Background**: Hospitalisation for paediatric oncohaematological diseases entails significant physical and psychological stress, often compromising children’s emotional regulation and daily functioning. In this context, complementary interventions can provide additional support to the clinical work conducted with children and adolescents undergoing treatment, fostering emotional awareness and well-being. This study evaluates the feasibility and perceived benefits of an animal-assisted psychoeducational intervention to enhance emotional coping during hospitalisation. **Methods**: A single-centre observational study was conducted at Regina Margherita Children’s Hospital in Italy in collaboration with the ZOOM Foundation (Turin, Italy), between September 2023 and May 2025. Sixty patients aged 6–15 years participated in an intervention combining observation of an in-ward aquarium, virtual animal encounters, and completion of a psychoeducational booklet promoting emotional reflection through symbolic identification with animal behaviours. **Results**: The intervention showed high feasibility and acceptability: 90% of participants (54/60) reported positive emotions and enjoyment, 80% (48/60) found the booklet engaging, and all participants (100%) perceived care and attention from facilitators. The activities fostered engagement, curiosity, and well-being, though their impact on deeper emotional processing appeared limited. **Conclusions**: Animal-assisted psychoeducational interventions are feasible and well-received in paediatric oncohaematology, offering complementary support to clinical care by promoting emotional resilience and enriching the hospital experience.

## 1. Introduction

Hospitalisation for oncohaematological diseases is a profoundly stressful experience for children, not only due to the physical demands of illness and treatment but also because of the emotional and psychological challenges it entails [1,2]. Beyond medical care, hospitalisation reshapes daily routines and the inner world of young patients. Emotions such as fear, curiosity, and hope coexist, and children may struggle to verbalise distress, making it crucial to provide symbolic spaces for expression to support emotional processing and coping. The clinical environment—often unfamiliar and intimidating—together with disruption of school, play, and social interactions, can generate feelings of isolation and helplessness. For many children, hospitalisation is marked by fear of medical procedures, uncertainty about the future, and a longing for normality. Prolonged stays, particularly in oncohaematology wards, can amplify these difficulties, affecting not only the child but also family dynamics. Addressing the psychological dimension of paediatric care is therefore essential—not merely as an optional supplement, but as a core component of comprehensive treatment. In recent years, there has been growing interest in integrative approaches that combine traditional medical care with supportive interventions aimed at enhancing emotional resilience [2]. Among these, Animal-Assisted Interventions (AAIs) have emerged as promising tools for promoting well-being in hospitalised children [3,4,5,6,7]. By facilitating meaningful interactions—direct or symbolic—with animals, AAIs can provide comfort, stimulate emotional processing, and foster a sense of connection and empathy [5,8]. Activities involving animals have been shown to improve mood and relieve anxiety in hospitalised patients and healthcare staff alike [4,6,9]. Engaging with animals can also create shared moments of attention and curiosity, offering children a sense of agency and participation even in constrained hospital environments. Observing wild animals and natural environments fosters empathy and conveys the calmness inherent in nature. Exposure to nature has been shown to restore physical, psychological, and emotional well-being, provide spaces that foster social support, and offer experiences that promote positive distraction and respect for the environment [10,11,12,13,14]. However, hospitalised children often face barriers to accessing natural environments due to limited mobility, chronic conditions, or compromised immune systems. To address this, in 2021, an aquarium containing dozens of African cichlid fish was installed in the Isola di Margherita ward of the Regina Margherita Children’s Hospital, which is dedicated to long-stay or end-of-life patients. The project aims to bring nature to all children who cannot access it, offering moments of distraction and enriching experiences that are as normal as possible during hospitalisation. The aquarium serves as a symbolic bridge, connecting children to the broader natural world and providing a visual and emotional anchor.

Beyond its psychoeducational purpose, the project also functions as a narrative and symbolic space, allowing children to reconnect with parts of themselves often silenced by illness.

This study explores the potential of an animal-assisted psychoeducational intervention to support children hospitalised for oncohaematological treatment. Through symbolic engagement with the animal world—via observation, storytelling, and reflection—the initiative seeks to create safe spaces for emotional exploration and promote adaptive coping mechanisms. The approach is grounded in scientific evidence and leverages the therapeutic benefits of nature and animals to strengthen emotional resilience, promote psychological well-being, and improve the overall hospital experience for patients [14,15,16]. In this context, the present study evaluates a single-centre observational study animal-assisted psychoeducational intervention developed at Regina Margherita Children’s Hospital in Turin, in collaboration with the ZOOM Foundation. The primary aims were to assess the feasibility and acceptability of the intervention in a paediatric oncohaematology setting and to explore children’s educational engagement and symbolic representations of coping. Emotional and psychological aspects were investigated at a descriptive and experiential level, rather than as formal clinical outcomes.

## 2. Methods

At Regina Margherita Children’s Hospital, in collaboration with the ZOOM Foundation, an initiative was launched integrating psychological support, educational activities, and creative resilience-building strategies for paediatric patients and their families. The project explores how interactions with animals—through observation, symbolic identification, and reflection on animal behaviours—can support emotional processing, foster emotional awareness, and promote adaptive coping mechanisms [17]. Grounded in Animal-Assisted Therapy (AAT), the intervention builds on evidence demonstrating its potential to reduce anxiety and emotional distress in paediatric hospital settings [2,18].

The initiative originated with the installation of an aquarium in the Isola di Margherita ward, offering young patients exposure to nature during prolonged hospital stays. A central component of the programme is a psychoeducational booklet containing experiential and reflective activities, including fish observation in the ward. For children unable to access the aquarium, online videos provided via QR codes ensured remote participation and engagement. These activities were designed to monitor emotional responses while assessing the intervention’s impact on the hospital experience.

Previous studies have highlighted the benefits of complementary therapies in enhancing paediatric psychological well-being [19]. This study extends that evidence by investigating whether emotional mirroring through interactions with the animal world can alleviate the emotional burden of hospitalisation, ultimately improving the quality of care for young patients.

The study employed a single-centre observational study at the Paediatric Oncohaematology Unit of Regina Margherita Children’s Hospital in Turin. Conducted from September 2023 to May 2025, it targeted paediatric patients with oncohaematological diseases, providing emotional support through psychoeducational activities focused on emotional mirroring via animal interactions.

The intervention consisted of interactive and experiential activities, including:

• One face-to-face group meeting per month, held in the hospital’s playroom. For immunological safety, meetings involved only a small number of participants, ensuring meaningful interaction.
• One online group meeting per month, conducted remotely to allow broader participation and maintain continuity of support outside the hospital.

All sessions were facilitated by trained psychologists and educators, ensuring emotional safety and adapting activities to children’s physical and emotional conditions. Data collected during the intervention included both quantitative and qualitative components.

Quantitative data derived from an enjoyment and feedback questionnaire were analysed descriptively using frequencies and percentages.

Qualitative feedback was collected from children’s verbal comments during sessions, written responses, and drawings produced as part of the activities. These materials were analysed using a thematic approach. All qualitative data were systematically reviewed to identify recurrent patterns related to emotional mirroring, symbolic identification with animals, perceived agency, curiosity, and engagement within the hospital context. To enhance reliability and reduce subjectivity, two trained psychologists independently examined the qualitative materials and subsequently discussed emerging themes until a shared interpretation was reached. Given the exploratory and observational nature of the study, this qualitative analysis was intended to provide descriptive insight into children’s lived experiences rather than to serve as a formal clinical assessment.

## 3. Procedure

In the ward, activities were conducted in both individual and group settings. One-on-one sessions took place in patient rooms, while small group sessions were held in the playroom. Each format presented distinct advantages and challenges. The group setting allowed for the simultaneous participation of multiple children, facilitating data collection, providing a recreational break and promoting peer socialisation. Challenges included heterogeneity in age among participants and, for some international patients, language barriers.

In addition to in-person activities, children participated in online sessions featured live observations of Zoom biopark animals. Led by a biologist, each session lasted approximately 60 min and included an interactive presentation followed by a question-and-answer segment. These online sessions enabled children to acquire knowledge about animals and engage with them symbolically.

During face-to-face group sessions in the Paediatric Oncohaematology playroom, the biologist guided children in completing the experiential booklet, titled “A Rainbow of Emotions: Play with Mbuna Fish and Discover Animals!”. The booklet comprises psychoeducational activities designed to facilitate exploration and reflection on emotions in relation to hospitalisation, drawing parallels with animal behaviour. Each session lasted approximately 90 min and included practical exercises and guided group discussions. At the conclusion of each session, children completed a satisfaction questionnaire to assess their experience.

The activities provided opportunities for children to express emotions that are often difficult to verbalise. Furthermore, they were effective in fostering a sense of agency and active engagement within the clinical setting.

## 4. Sample

The study involved paediatric oncohaematological patients admitted to Regina Margherita Children’s Hospital.

Inclusion criteria:

• Diagnosis of oncohaematological disease.
• Age between 6 and 15 years.
• Provision of written informed consent (obtained from parents or legal guardians for minors).

Exclusion criteria:

• Lack of informed consent.
• Inability to comprehend the study objectives.

A total of 60 paediatric oncology patients were recruited over a period of one-and-a-half years (mean age = 9.3 years). Of these, 26 were male (43.3%) and 34 were female (56.7%). The median age was 9.7 years for males and 8.9 years for females. Regarding diagnosis, 70% of patients had leukaemia or lymphomas, 15% had solid tumours, and 15% had brain tumours (Table 1).

**Table 1:** Sample characteristics of paediatric oncohaematology patients included in the study. (N = 60) ^1^.

Department of Oncohaematology	Sex	Mean age	Total *n*
	M	9.7	26
	F	8.9	34
TOT.		9.3	60
Diagnosis			
Leukaemia/Lymphoma	42 (70%)		
Solid tumours	9 (15%)		
Brain tumours	9 (15%)		

^1^ Mean age is presented in years. Totals include all patients enrolled in the study.

## 5. Tools

Each participant received a psychoeducational booklet entitled “A Rainbow of Emotions: Play with Mbuna Fish and Discover Animals!”. Mbuna, the guiding fish, accompanies children through the various planned activities. The booklet was specifically designed to foster emotional awareness and regulation, through playful and reflective exercises centred on observing and interacting with animals.

### 5.1. Structure and Content of the Booklet

The booklet introduces the primary emotions—joy, sadness, anger, fear, disgust, and surprise—which shape children’s inner experiences and enable them to relate to themselves and others. Through observation of animals and their behaviours, children are encouraged to reflect on their own emotional experiences. The booklet guides participants in recognising and naming their feelings, drawing parallels between emotions and animal behaviours, and engaging in playful exercises that promote emotional awareness, self-regulation, and social interaction [5].

By combining observation, reflection, and hands-on activities, the booklet offers a meaningful and enjoyable method for children to explore and express emotions during hospitalisation. The activities include:

• Drawing-based activities that invite children to project aspects of their emotional world through symbolic representation. In particular, they are encouraged to depict animals with which they feel an emotional affinity, or to illustrate how they would wish to be if they were an animal. This creative process facilitates the expression of inner experiences, desires, and self-perceptions, offering valuable insights into the child’s emotional and relational functioning.
• Observation-based reflective activities that engage participants in the analysis of animal behaviour—in this case, fish—with the aim of fostering self-awareness and emotional insight. By observing and interpreting the animals’ movements, interactions, and responses to environmental stimuli, participants are encouraged to draw parallels between these behaviours and their own emotional experiences and regulatory strategies.
• Role-playing activities designed to engage children in reflective thinking about animal adaptations and their possible parallels in human functioning. Through these exercises, children are invited to identify which animal’s characteristics they would wish to possess in order to enhance their own adaptation to the surrounding environment—for example, the ability to live within a cohesive group where cooperation ensures protection, or to conceal themselves effectively in challenging situations. They are also encouraged to recognise and select the behavioural traits and adaptive strategies that they perceive as most representative of themselves, such as heightened visual or auditory sensitivity, or the capacity for camouflage. Corresponding pages from the experiential workbook will be included at the end of the article, by way of illustration, to exemplify the proposed activities and their practical application (Supplementary Files 1 and 2)

Each activity is accompanied by clear, child-friendly instructions and visual aids to enhance accessibility and engagement. These exercises facilitate articulation and reflection on emotions that are often difficult to verbalise, while promoting engagement and emotional agency.

### 5.2. Accessibility and Multimedia Support

For children unable to visit the “Isola di Margherita” aquarium, the booklet includes a QR code containing links to online videos of the fish, thus enabling remote participation. The accessibility and functionality of the digital content were confirmed, with no significant technical issues reported during implementation.

### 5.3. Evaluation and Feedback

Upon reaching the end of the booklet, children completed an enjoyment and feedback questionnaire to assess their engagement and the perceived benefits of the activities. The questionnaire consisted of eight closed-ended questions rated on a 5-point Likert scale (e.g., “How much did you enjoy the activity?”, “Did you find the booklet interesting?”, “Did the activities reduce hospital-related stress?” ranging from 0 (“not at all”) to 5 (“extremely”). Feedback was analysed both quantitatively and qualitatively to capture children’s subjective experiences, identify recurring themes, and inform future adaptations of the intervention.

## 6. Results

Results of the Enjoyment and Feedback Questionnaire, addressing participants’ emotional experience, engagement with the activities, and perceived impact on the hospital experience, are summarised in Table 2.

Overall, participants (N = 60) responded positively to the activities. Most children reported a positive emotional state during the activities, with 75% responding “Good” and 15% “Very good.” Interest in the booklet was similarly high, with 80% of participants expressing positive engagement (“Very much” or “Yes”). Enjoyment of the activities was reported by 90% of children, and an equal proportion (90%) reported acquiring new knowledge about the animal world.

In contrast, only a minority of participants (20%) reported gaining new insights into their own emotions, while the majority (60%) were uncertain about this. This result is consistent with the design of the booklet, which aimed primarily to enhance emotional attunement with the animal world rather than to foster personal emotional insight.

The most frequently reported emotions during the activities were joy 25% (15/60), anger 23% (14/60), surprise 20% (12/60), and sadness 17% (10/60). All participants reported feeling cared for and supported by healthcare professionals, with 100% of responses indicating satisfaction. This finding is particularly relevant, as it reflects the perceived quality of the relational environment and the effectiveness of supportive interactions during the activities.

Regarding the impact of the activities on the daily hospital routine, responses were more varied: 15% reported a positive effect, 60% reported a neutral effect, and 25% reported a negative effect.

**Figure 1:**
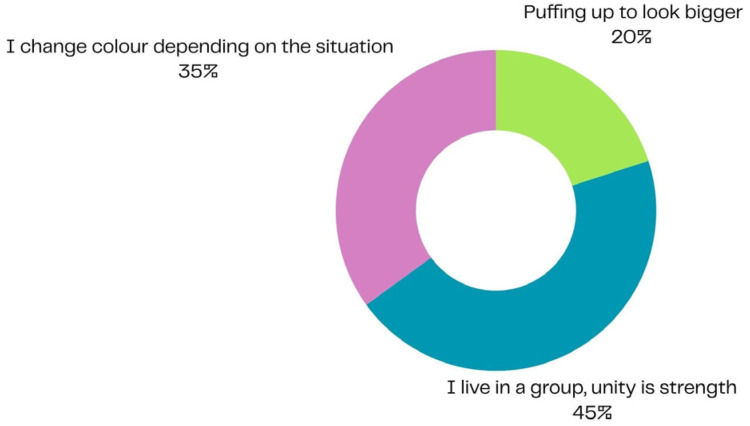
Adaptive behaviours and characteristics reported by children as helpful for coping with the hospital environment, reflecting symbolic strategies of protection, flexibility, and relational support (N = 60; percentages correspond to 27, 21, and 12 participants, respectively).

**Figure 2:**
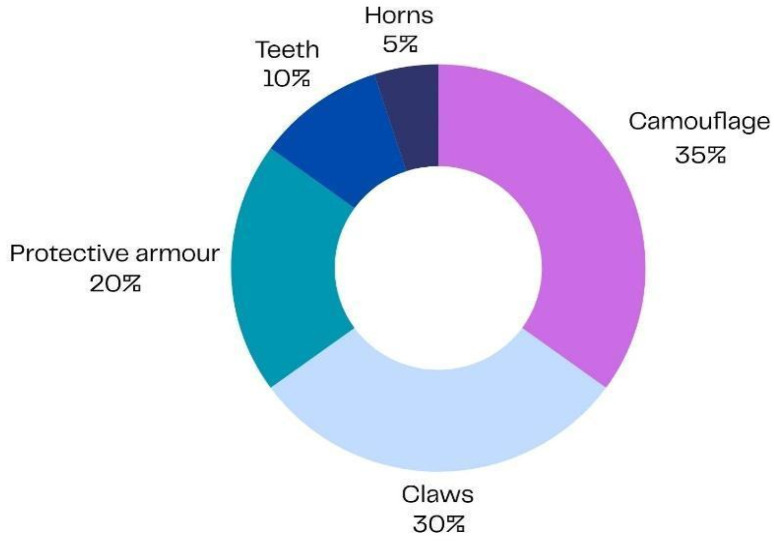
Physical and behavioural adaptations identified by children as helpful for coping with hospitalisation, reflecting symbolic needs (N = 60; percentages correspond to 21, 18, 12, 6, and 3 participants, respectively).

In line with the study’s aim of exploring children’s perceived coping strategies and symbolic resources activated during hospitalisation, the following findings describe the behavioural and physical characteristics of animals selected by participants through a process of symbolic identification as helpful for adapting to the hospital environment (Figure 1 and Figure 2).

Another interesting finding concerns the main responses given by patients regarding the behaviours they would like to adopt to better adapt to their environment. The most frequent responses—“I live in a group, unity is strength,” “I change colour depending on the situation,” and “I puff up to look bigger”—meaningfully reflect the adaptive needs and coping strategies of paediatric patients with oncological and haematological conditions.

Approximately 45% of participants (27/60) chose the option related to living in a group, implicitly expressing the importance attributed to relational support and cohesion with family members, peers, and healthcare staff—key elements in coping with the experience of illness.

About 35% (21/60) selected the ability to change colour depending on the situation, suggesting a tendency toward flexible adaptation but also a need to modulate emotions and behaviours according to a context often perceived as unpredictable or anxiety-provoking.

Finally, around 20% (12/60) chose the strategy of “puffing up to look bigger”, which may symbolically represent a defensive stance and an attempt to regain a sense of control—a way to feel strong and competent when faced with vulnerability and fear (Figure 1).

Overall, these responses show how, even within playful and symbolic activities, children communicate essential needs for safety, connection, and self-confidence while navigating the challenges of illness.

With regard to physical and behavioural adaptations, the most frequently selected characteristics were camouflage abilities, claws, and protective armour, followed by teeth and, to a lesser extent, spines and horns.

Approximately 35% of participants (21/60) chose camouflage, reflecting a wish to blend into the environment and protect themselves by becoming less visible in stressful or unpredictable situations. Around 30% (18/60) selected claws, symbolising determination, action, and the capacity to face challenges directly. Another 20% (12/60) opted for protective armour, expressing a need to feel safe, shielded, and emotionally contained within the hospital setting.

About 10% of children (6/60) opted for teeth, often associated with strength, power, and self-defence—a way of symbolically expressing a desire to regain a sense of control and agency.

Finally, a small proportion, approximately 5% (3/60), selected spines or horns, traits that may represent additional protective strategies and the wish to establish clear personal boundaries (Figure 2).

Taken together, these findings highlight the diversity of adaptive themes expressed by the children—ranging from protection and invisibility to strength and assertiveness—offering valuable insight into how they imagine coping with the psychological and emotional challenges associated with illness and treatment.

## 7. Discussion

The findings of this study indicate that the psychoeducational booklet was considered interesting by 80% of participating children (20% “Very much” (12/60), 60% “Yes” (36/60). The planned activities effectively supported the acquisition of new knowledge—particularly regarding the animal world, as reported by 90% of participants (54/60)—and, to a lesser extent, facilitated emotional awareness, with only 20% of children (12/60) reporting new insights about their own emotions, and 60% uncertain (36/60). This outcome is consistent with the booklet’s design, which aimed primarily to enhance emotional attunement with animals rather than introspection on personal feelings [5].

The intervention provided symbolic spaces for expressing emotions that are often difficult to verbalise during hospitalisation. The children described animals as companions capable of understanding feelings without the need for words. Observing the aquarium or interacting with the booklet allowed them to “feel outside the hospital for a while,” Taken together, these experiences should be interpreted primarily as indicators of feasibility, acceptability, and perceived emotional experience during the intervention, rather than as evidence of changes in emotional regulation or psychological functioning.

Both qualitative and quantitative data suggest that the intervention was well received. Most children reported positive emotional states during activities (75% “Good,” 15% “Very good”) and high enjoyment levels (90%), indicating positive emotional experiences during the activities and high levels of enjoyment. Frequently reported emotions included joy, anger, surprise, and sadness, reflecting a broad range of emotional engagement. All participants (100%) reported feeling cared for and attended to by facilitators, underscoring the importance of perceived emotional support in paediatric hospital settings. This perception is a key factor for engagement, emotional expression, and overall well-being, suggesting that the intervention functioned primarily as an educational and relational support, fostering a supportive and trusting environment rather than a structured psychological intervention.

In-person sessions with facilitators and the external educator were essential components of the intervention. These encounters enabled children to engage with the booklet while experiencing curiosity, exploration, and discovery. Through discussions, observations, and guided interactions with animals and the aquarium, children connected with aspects of the natural world typically inaccessible during hospitalisation. Even within the hospital environment, these activities provided a symbolic window into the broader world, allowing children to shift their attention beyond their rooms and engage imaginatively with life outside the hospital.

For many children, observing animals, drawing, or completing the booklet with the guidance of a supportive adult became a vehicle for cognitive and emotional engagement. The activities allowed them to express feelings indirectly, reflect on experiences, and feel competent, even in contexts that might otherwise limit autonomy. The combination of curiosity-driven learning and emotional support contributed to a richer hospitalisation experience, offering a brief escape from routine medical care while maintaining support and understanding.

Regarding the perceived impact on daily hospital routines, 15% of participants (9/60) reported a positive effect, 60% a neutral effect (36/60), and 25% (15/60) a negative effect. This suggests that while activities alleviated boredom for some children, they primarily served as a low-threshold supportive complement rather than a replacement for structured psychological care. Completing the booklet in patients’ rooms also fostered shared experiences between children and caregivers. For activities addressing more emotional content, the presence of an external educator facilitated recognition and expression of feelings.

Overall, these findings support the feasibility and acceptability of interactive, educational, and play-based tools in supporting engagement, curiosity, and emotional attunement in paediatric hospital settings. Importantly, the results highlight that the human dimension of facilitation—the perception of care, attention, and relational support—constitutes a central determinant of intervention effectiveness, reinforcing the value of integrating these activities with formal psychological support to enhance engagement, curiosity, and emotional attunement.

## 8. Limitations

This study has several limitations that should be acknowledged. Firstly, it was a single-centre observational study with a relatively small sample size (N = 60), which may limit the generalisability of the findings to other paediatric settings or populations with different cultural, organisational, or clinical characteristics, as well as the statistical power of the conclusions. Engagement with children in the Isola di Margherita ward proved challenging, as many participants were often too fatigued to take part in activities.

Secondly, the observational design does not allow for causal inferences regarding the relationship between the intervention and the reported emotional or learning benefits. Although participants described positive experiences, the absence of a control group prevents attributing these outcomes solely to the psychoeducational intervention.

Thirdly, evaluation relied primarily on self-report measures and a brief feedback questionnaire administered at the end of the activities. While these tools offered valuable insights into children’s experiences, they are subject to potential biases, including social desirability and limited self-awareness, particularly among younger participants.

Additionally, heterogeneity in age, medical condition, and language proficiency may have influenced how activities were perceived and experienced. For instance, older children tended to engage more willingly in individual in-person sessions than group meetings, whereas younger participants often perceived the workbook as a task to complete. Language barriers further challenged foreign patients’ participation and comprehension. Future research could benefit from stratifying participants by age or clinical status and designing materials tailored to the developmental needs of different age groups.

Moreover, the short-term nature of the intervention and lack of follow-up data prevent conclusions about its long-term psychological impact or coping strategies.

It is also important to emphasise that the proposed activities are intended as an additional resource and do not replace standard psychological or educational interventions provided within the hospital. They serve as complementary support to enhance patient engagement, promote emotional expression, and alleviate the psychological burden of hospitalisation in a creative and non-invasive way.

Future studies could integrate participatory methodologies, encouraging children to co-design activities and reflect on their own emotional narratives, thereby enhancing engagement and the interpretive richness of findings. Despite these limitations, the results provide preliminary evidence of the feasibility and acceptability of an animal-assisted psychoeducational approach in paediatric oncology, laying the foundation for further research using more rigorous methodologies.

## 9. Conclusions

This study provides preliminary evidence that an animal-assisted psychoeducational booklet represents a feasible and well-received intervention in paediatric hospital settings. The activities facilitated engagement, enjoyment, and learning—particularly regarding the animal world—while offering symbolic spaces for emotional expression. Beyond the booklet itself, interactions with the biologist were essential, enabling children to engage directly, ask questions, and explore new curiosities about animals. These interactions not only promoted relational connection and playful exploration but also supported children’s empowerment, fostering a sense of agency and active participation in their hospital experience.

Importantly, all participants reported feeling cared for, highlighting the relevance of both emotional and physical support in paediatric hospital care. While the booklet had a limited effect on personal self-reflection, it contributed to improved mood and psychological well-being, underscoring the value of complementary, play-based approaches alongside standard psychological interventions. Interactive educational tools such as this can reduce hospital monotony, stimulate curiosity, enhance emotional attunement, and foster a sense of competence and autonomy in hospitalised children.

Future research should focus on larger, multicentre studies, the development of age- and condition-tailored activities, and participatory designs to further evaluate feasibility and acceptability, while also exploring potential effects on emotional awareness, coping strategies, and child empowerment within hospital settings.

## Figures and Tables

**Table 2:** Descriptive results of the Enjoyment and Feedback Questionnaire assessing feasibility and acceptability of the animal-assisted psychoeducational intervention (N = 60).

Area of Investigation	Main Responses (%)
Perceived emotional state *“How do you feel?”*	Very good 15% (9/60) Good 75% (45/60) Average 10% (6/60)
Interest in the booklet *“Did you find the booklet interesting?”*	Very much 20% (12/60) Yes 60% (36/60) Average 20% (12/60)
Enjoyment of activities *“Did you enjoy the activities?”*	Yes 90% (54/60) Not sure 10% (6/60)
Discovery of new information about the animal world *“Did you discover something new about the animal world?”*	Yes 90% (54/60) No 5% (3/60) Not sure 5% (3/60)
Discovery of new aspects about one’s emotions *“Did you discover something new about your own emotions?”*	Yes 20% (12/60) No 10% (6/60) Not sure 60% (36/60)
Most frequent emotion experienced *Which emotion was reported most frequently?*	Joy 25% (15/60), Anger 23% (14/60), Surprise 20% (12/60), Sadness 17% (10/60), Fear 10% (6/60), Disgust 5% (3/60)
Perception of care and attention from facilitators *“Did you feel that we took care of you?”*	100% positive (60/60)
Impact of activities on daily hospital routine *“Did the activities make hospital days less boring?”*	Positive 15% (9/60) Neutral 60% (36/60) Negative 25% (15/60)

Some children were supported by an external educator during the completion of the questionnaire. Responses were collected using pictorial Likert scales with emotive facial icons to facilitate comprehension and expression among younger participants.

## Data Availability

The data presented in this study are available on request from the corresponding author. The data are not publicly available due to privacy.

## Supplementary Materials

The following supporting information can be downloaded at: https://www.mdpi.com/article/10.3390/children13010136/s1. File S1 legend: Animal Adaptive Behaviors and Distinctive Characteristics. File S2 legend: Animal Protective Adaptations.
