# Supplementary material for: Animal-Assisted Psychoeducational Intervention in Paediatric Oncohaematology: Evidence from a Single-Centre Observational Study
Source: Children (Basel). 2026 Jan 16;13(1):136. doi: 10.3390/children13010136 (PMC12840532; doi:10.3390/children13010136)

Supplementary File S1

Animal Behaviour

The work of an ethologist is truly fascinating because every species has its own unique physical and behavioral characteristics. These traits help animals adapt better to their environment and improve their chances of survival. Some have sharp claws used for hunting, while others use them to climb and escape. Some pretend to be dead to trick predators and avoid being eaten; some choose to live in groups, while others prefer a solitary life. Some puff themselves up or spread their limbs to look bigger and more frightening, while others make themselves very small and hide inside shells or burrows. There are even animals that can change colour to blend in with their surroundings and avoid being seen.

Answers: 1) LEOPARD: I live in a group—unity is strength; 2) PUFFERFISH: I puff myself up to look bigger: 3) OCTOPUS: I change colour depending on the situation; 4) SNAKE: I'm coloured to resemble something more dangerous—even though I'm not; 5) FISH: I protect my eggs inside my mouth; 6) HERMIT CRAB: I'm good at hiding; 7) RHINOCEROS: I'm not afraid, because I'm big and strong, and I move toward danger. 8) TIGER: I live alone—if you want something done well, do it yourself

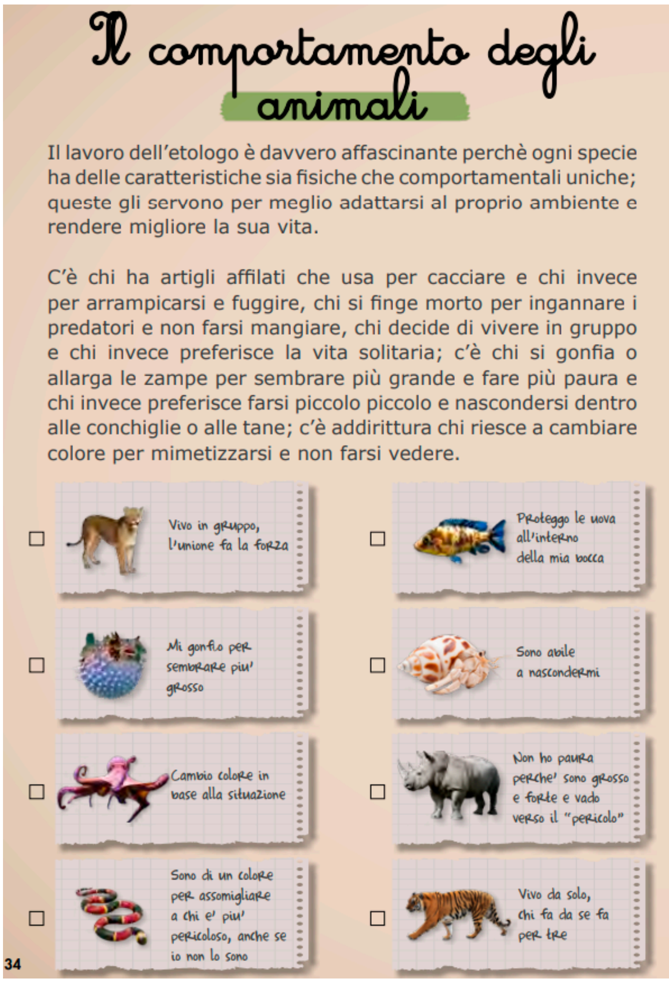

Il comportamento degli animali

Il lavoro dell'etologo è davvero affascinante perchè ogni specie ha delle caratteristiche sia fisiche che comportamentali uniche; queste gli servono per meglio adattarsi al proprio ambiente e rendere migliore la sua vita.

C'è chi ha artigli affilati che usa per cacciare e chi invece per arrampicarsi e fuggire, chi si finge morto per ingannare i predatori e non farsi mangiare, chi decide di vivere in gruppo e chi invece preferisce la vita solitaria; c'è chi si gonfia o allarga le zampe per sembrare più grande e fare più paura e chi invece preferisce farsi piccolo piccolo e nascondersi dentro alle conchiglie o alle tane; c'è addirittura chi riesce a cambiare colore per mimetizzarsi e non farsi vedere.

- ☐ Vivo in gruppo, l'unione fa la forza
- ☐ Mi gonfio per sembrare piu' grosso
- ☐ Cambio colore in base alla situazione
- ☐ Sono di un colore per assomigliare a chi e' piu' pericoloso, anche se io non lo sono
- ☐ Proteggo le uova all'interno della mia bocca
- ☐ Sono abile a nascondermi
- ☐ Non ho paura perche' sono grosso e forte e vado verso il "pericolo"
- ☐ Vivo da solo, chi fa da se fa per tre

34

Supplementary File S2

In general, the physical and behavioral adaptations of animals serve as defensive weapons—to avoid being eaten—or offensive tools, to catch their prey. Choose one behaviour and one adaptation from the list below by circling the ones that best represent you:

-Spines

-Super-sight

-Claws

-Horns

-Super-hearing

-Teeth

-Camouflage that blends in with the environment

-Armour

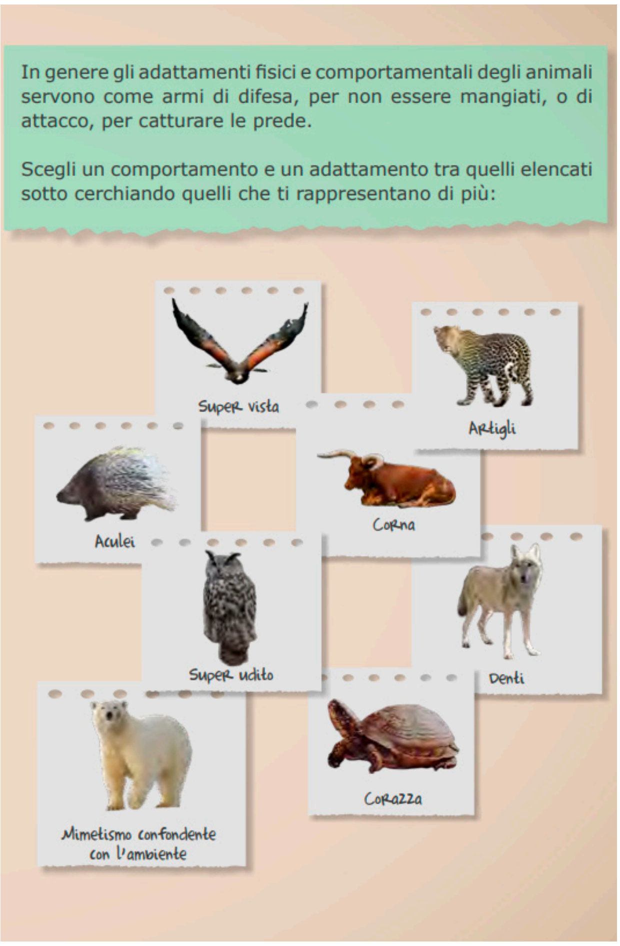

Supplement: Supplementary file 1 [file children-13-00136-s001.zip › children-4034753-supplementary.pdf]
